# Green Toxicology: a strategy for sustainable chemical and material development

**DOI:** 10.1186/s12302-017-0115-z

**Published:** 2017-04-04

**Authors:** Sarah E. Crawford, Thomas Hartung, Henner Hollert, Björn Mathes, Bennard van Ravenzwaay, Thomas Steger-Hartmann, Christoph Studer, Harald F. Krug

**Affiliations:** 1 Institute for Environmental Research, RWTH Aachen University, Worringerweg 1, 52074 Aachen, Germany; 2 John Hopkins University, Bloomberg School of Public Health, 615 N. Wolfe Street, Baltimore, MD 21205 USA; 3 CAAT-Europe, University of Konstanz, Universitaetsstrasse 10, 78467 Constance, Germany; 4 DECHEMA e.V., Theodor-Heuss-Allee 25, 60486 Frankfurt, Germany; 5 BASF SE, Carl-Bosch-Straße 38, 67056 Ludwigshafen, Germany; 6 Investigational Toxicology, Bayer AG, Müllerstraße 178, 13352 Berlin, Germany; 7 Federal Office of Public Health, Schwarzenburgstraße 157, 3003 Bern, Switzerland; 8 Empa, Materials Science and Technology, Lerchenfeld-straße 5, 9014 St. Gallen, Switzerland

**Keywords:** Green Toxicology, Green Chemistry, Predictive toxicology, Alternative animal testing, In vitro assays, Toxicogenomics

## Abstract

Green Toxicology refers to the application of predictive toxicology in the sustainable development and production of new less harmful materials and chemicals, subsequently reducing waste and exposure. Built upon the foundation of “Green Chemistry” and “Green Engineering”, “Green Toxicology” aims to shape future manufacturing processes and safe synthesis of chemicals in terms of environmental and human health impacts. Being an integral part of Green Chemistry, the principles of Green Toxicology amplify the role of health-related aspects for the benefit of consumers and the environment, in addition to being economical for manufacturing companies. Due to the costly development and preparation of new materials and chemicals for market entry, it is no longer practical to ignore the safety and environmental status of new products during product development stages. However, this is only possible if toxicologists and chemists work together early on in the development of materials and chemicals to utilize safe design strategies and innovative in vitro and in silico tools. This paper discusses some of the most relevant aspects, advances and limitations of the emergence of Green Toxicology from the perspective of different industry and research groups. The integration of new testing methods and strategies in product development, testing and regulation stages are presented with examples of the application of in silico, omics and in vitro methods. Other tools for Green Toxicology, including the reduction of animal testing, alternative test methods, and read-across approaches are also discussed.

## Background

Over the past two decades, the movement of Green Chemistry has become a new standard embraced for the development of less harmful materials and chemicals that are safer for both the environment and consumers [1, 2]. The twelve principles of Green Chemistry outlined by Anastas and Warner [3] and mnemonically presented by Tang et al. [4] (PRODUCTIVELY; prevent wastes; Renewable materials; omit derivatization steps; degradable chemical PRODUCTS; use safe synthetic methods; catalytic reagents; temperature, pressure ambient; in-process monitoring; very few auxiliary substances; E-factor, maximized feed in products; low toxicity of chemical products; yes, it’s safe) are a guideline to develop less-hazardous products through safer methods that minimize harmful waste and exposure (i.e. benign-by-design). Many of these goals, along with the principles of Green Engineering [4], strive for sustainability with chemical synthesis and molecular design [2] and are adopted by major industries (e.g. pharmaceutical and chemical). However, currently and for the future, the inclusion of aspects related to consumer and environmental health has become more and more important. Thus, considerations about the possible toxic activity of a certain molecule or material during its development for the market are crucial not only for the economic success but also for its consumer acceptance. Taking this aspect into account, Green Toxicology will strengthen the marketing process and avoid serious setbacks.

Green Chemistry practices have been adopted into mainstream research and manufacturing since the early 1990s. Success stories of the application and study of Green Chemistry include the use of microbes as environmentally benign synthetic catalysts [5–7] as well as the development of fully biodegradable bags with the use of compostable polyester film (e.g. Ecoflex^®^; [8–10]). The efforts of Green Chemistry have resulted in the reduction of hazardous waste in a cost-effective manner that has maintained the need, efficacy and safety of products for consumers. For example, the development of water-based acrylic alkyd paints with low volatile organic carbons (VOCs) from recycled soda bottle plastic (polyethylene terephthalate; PET), acrylics and soybean oil reduced approximately 350,000 kg of VOCs during manufacturing in 2010 [11]. Thus, Green Chemistry practices are now incorporated into higher education, prominent at scientific conferences and in journals, and are easily recognized by the industry for their advantageous benefits commercially, environmentally and publicly [12, 13]. However, toxicity, which is a large part of risk and hazard assessments, is not intrinsically considered in the Green Chemistry and Green Engineering approaches. For example, the use of solvents, which consistently account for approximately 80–90% of the materials used in a typical pharmaceutical batch chemical operation [14], may play a dominant role in the overall toxicity of any given manufacturing process, resulting in excess and potentially hazardous waste. Due to the sometimes irreplaceable utility of solvents, it is vital that the toxicological hazards of all aspects (health, safety, and lifecycle) of solvent selection, use, interaction, processes and disposal be evaluated.

**Fig. 1:**
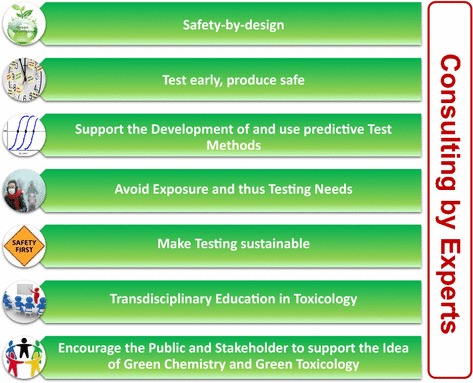
Principles of Green Toxicology

A complementary tool for Green Chemistry and Green Engineering that incorporates the toxicological risk and hazard assessment of the design to disposal of products and materials is the concept of Green Toxicology. Green Toxicology [15] describes the application of predictive toxicology in the design, manufacturing, use and disposal of new materials and chemicals. The objective of such an application is to contribute to products, which are safer for humans and the environment by using intelligent and predictive testing strategies of toxicology. Maertens et al. [1] outlines several considerations, which might form the basis of future principles of Green Toxicology, the basis of which are: (1) benign-by-design (also known as safety-by-design); (2) test early—produce safe; (3) avoid exposure and thus testing needs; and (4) make testing sustainable. These considerations of Green Toxicology are outlined in Fig. 1, which encompass the fundamental ideas of Green Chemistry, but which additionally utilize predictive toxicological testing tools and strategies. In other words, Green Toxicology aims to expand the respective principles of Green Chemistry to develop and produce products that are less toxic, with safer processes that result in less waste and exposure, utilizing toxicological tools and strategies. While there are many overlapping features and principles among the Green Chemistry, Engineering and Toxicology, the key difference of Green Toxicology is that it promotes the incorporation of toxicological considerations throughout the discovery, development, and production of new materials and chemicals, which are discussed in this paper.

Toxicity testing is a prerequisite for reducing the risks to humans and the environment. However, sustainable practices, which are the pillars of Green practices, require the reduction in not only animal testing but of the chemicals used during toxicity testing. Green Toxicology practices promote and encourage the use of new and innovative techniques and strategies that reduce the use of animals for testing, the amounts of chemicals used and disposed of during tests; and increase the consideration of toxicity in the synthesis, use and regulation of chemicals. Frontloading of toxicity assessments (i.e. not waiting until regulatory or market release until safety assessments have to be done) by means of intelligent and predictive testing strategies are key to the Green Toxicology approach. This implies that, often, many more substances still under consideration have to be assessed, and that quantities of substances and resources for testing are limited. These limitations call for the use of computational and higher-throughput in vitro approaches. In such a way, Green Toxicology becomes an integrated component of the Green Chemistry approach in that an understanding of the adverse outcomes and associated toxicity of chemical development, use, and disposal are recommended. Toxicological tools, such as in silico, omics and in vitro methods allow for a better understanding of the mechanisms of toxicity, identification of common structural activity relationships (SAR) and associated effects, and thus, can often eliminate potential chemical candidates early on based on predicted toxicity (e.g. “failing early and failing cheaply”).

Green Toxicology offers many advantages in the practices and application of Green Chemistry, which are discussed in this paper. The Green Toxicology principles outlined by Maertens et al. [1] and Fig. 1 provide a framework for designing chemicals that are safer for humans and the environment by utilizing new and innovative predictive toxicological tools and strategies. This paper discusses some of the aspects of Green Toxicology with respect to improving the integration of Green Toxicology with Green Chemistry practices to produce safer, less harmful products. The integration of new testing methods and strategies in product development, testing and regulation stages are presented with examples of the applications of in vitro, omic, and in silico methods. Other tools for Green Toxicology, including the reduction of animal testing, alternative test methods, and read-across approaches are also discussed. Examples of lessons that can be learned from past activities are also discussed with respect to reducing current and future risks (e.g. late lessons from early warning; precautionary principle; [16–18]). This paper also examines some of the stages of product development, regulation, use and disposal that can or have benefited from the incorporation of Green Toxicology practices. In addition, some of the most relevant aspects, advances and limitations of the emergence of Green Toxicology from the perspective of different industry and research groups will be discussed.

## Integration of Green Toxicology in discovery, development and production practices

In order to efficiently develop new compounds or products with the desired technological or biological traits and lesser toxicity, many different structures need to be evaluated. Synthesizing these new molecules, often with complex chemical structures, in sufficient amounts is very demanding. The discovery and development of new products and active ingredients (AIs) thus rely on reaction screening and route scouting with high-throughput experimentation incorporating automated solid and liquid handling for rapid and routine screening (e.g. evaluate typically 100 reactions simultaneously at 1 mL scale) [19]. Principle design is incorporated in all aspects of the AI process, including reaction screening, optimization, critical parameter identification and process response surface modelling [19]; however, the toxicity of all steps of chemical development and processing (e.g. intermediates, solvents, catalysts, etc.) must be considered.

Identifying the anticipated biological traits of AIs and products often requires only one assay, as the target is known. In contrast, the “off-target” specification (reduced toxicity) requires many tests, including those with animals. The reduction of animal testing is another pillar of ethical-based sustainability. Animal testing enables the assessment of toxic effects and thus helps to develop less toxic compounds. Animal testing is actually performed rather late during the development of new chemicals. However, even a very limited safety testing programme, according to the Organisation for Economic Co-operation and Development (OECD) guidelines, would require up to 0.5 kg of the compound. There are a number of other negative aspects associated with animal testing including animal welfare and ethics, as well as the heavy reliance and use of time (months to years per assay), money (thousands to millions of dollars per testing programme) and resources [15, 20]. In addition, the uncertainty associated with the extrapolation from animals to humans may be accounted for using uncertainty or safety factors; however, many variables such as body weight, homology of genetic material and enzymes, as well as anatomy and physiology of organisms and humans must be considered to make confident conclusions about human hazard assessments from animal data [15, 21].

Animal testing will, for the foreseeable future, be an essential and compulsory step in assessing the risk and regulation of chemical development, as various industrial sectors have different legal constraints placed upon them as consequences of registration and approval procedures worldwide. However, new tools and Green Toxicology strategies to relate mechanistic information obtained through in silico, toxicogenomic and in vitro approaches for animal and human effects have been investigated as alternative or complementary tools for in vivo tests. For example, with the development of alternative in vitro methods, test substance demand is generally reduced to less than 500 mg per assay. Hence, with only a few grams of a new compound, many tests can be performed. The use of predictive toxicological tools and strategies in Green Toxicology practices are discussed below.

### Predictive toxicology using in silico tools

In silico toxicology relies on the use of computational methods to analyse, model, and predict the toxicity of chemicals, which complement traditional and innovative toxicity tests for risk and hazard assessments. The use of in silico tools not only improve predictive toxicology, but also contributes towards the prioritization of chemicals, provides insight into future toxicity tests, and minimizes late-stage failures in chemical design (i.e. test early, produce safe). These advantages all aid in the incorporation of Green Toxicology in the development of new chemicals and products that benefit both the environment and consumers. In silico tools like the United States Environmental Protection Agency’s (U.S. EPA’s) ToxCast™ [22], part of the Toxicology in the twenty first century (Tox21), is a publicly available high-throughput toxicity dataset of thousands of chemicals that should be better utilized for data mining to advance Green Toxicology efforts in product development. Pertinent data from such in silico tools can provide crucial insight early on in the design process based on access to hundreds of measured and modelled assays used to screen chemicals, concentration–response curves, animal toxicity studies, and endocrine disrupting screening programmes that can advance Green Toxicology efforts [22, 23].

Similar success for Green Toxicology efforts can arise from the use of (quantitative) structural activity relationships [(Q)SARs], the use and applicability of which may lead to more successful Predictive Toxicology. QSARs are a predictive tool that provide insight into the potential toxicity of chemicals based on patterns of structure–function relationships from similar chemicals whose activities have already been assessed. Successful use of QSARs requires a sufficient amount of input data in order to support structure–toxicity relationships for risk assessment. A number of computerized models have been developed and improved to accommodate large datasets from high-throughput screening efforts (i.e. ECOSAR; Toxicity Estimation Software Tool; TEST; [24, 25]). As outlined by Anastas [15], QSAR approaches have been successfully used for the risk assessments of a number of chemicals including dioxins and furans, polychlorinated biphenyls (PCBs) and polyaromatic hydrocarbons (PAHs). However, there are a number of limitations of rule-based SAR and the correlative QSAR approaches [26]. Both are limited by the availability and quality of input data (“trash in, trash out”). Empiric rules are often derived from rather few compounds and not evaluated against large sets of chemicals. In addition, correlative approaches based on multiple chemical descriptors often suffer from over-fitting of parameters, especially when the number of parameters and chemicals of the training set are not proportionate. It is important to note that up until now no such approach has been internationally validated with chemical sets, which were not part of the training set (external validation), and broadly accepted.

Additional challenges for the use of in silico methods are that toxicological studies, to a large extent, are done by the industrial sector, and thus are used for registration as proprietary data and are not typically published or publicly available. This is especially true for the high-quality, standardized test data produced according to international test guidelines under quality assurance schemes such as Good Laboratory Practice (GLP). There was a pivotal change in the sharing and availability of such data, when for the first time, the European REACH legislation mandated that dossier summaries be published, making data readily available on a new scale. While this, together with the enormous testing demands of REACH [27, 28], stimulates the development of in silico approaches, the actual availability via the website of the European Chemical Agency (ECHA) is cumbersome and not machine readable. At the same time, REACH has made study data a commodity as owners have to be reimbursed by other registrants, which has lowered the willingness to freely share data. In addition, an approach that aggregates the use of data for in silico approaches, which does not require legitimate access to study dossiers, is lacking. However, making these data available for the OECD (Q)SAR Toolbox [29] and the European Chemical Industry Council’s (CEFIC) AMBIT tool [30] signals a step towards data interpretation by ECHA. In order to make these most valuable data more broadly available, the information was downloaded in its entirety, organized in a database and made machine readable by natural language processing [31]. This new database, paralleled by the creation and release of various other smaller databases with legacy data, which will be combined in the future, enables the analysis of the chemical landscape, the assessment of performance of traditional tests, and the development of new tools [32]. Early examples demonstrate such uses for oral acute toxicity [33], eye irritation [34] and skin sensitization [35].

Furthermore, the grouping of substances and read-across approaches were developed for filling data gaps in registrations of chemicals. Read-across approaches utilize weight-of-evidence approaches to make use of shared properties of an untested compound to a known compound. While sharing many characteristics of a QSAR, read-across approaches do not seek a mathematical formula for larger parts of the chemical universe but instead are based on “local” similarity and shared properties of chemicals [36, 37]. In addition to the similarity in the structure and physicochemical properties, biological data are also used to compare biological similarity among chemicals in read-across approaches. Good Read-Across Practices (GRAPs) were created and developed by the European Registration, Evaluation, Authorisation and Restriction of Chemicals (REACH) legislation as a result of the broad use of read-across approaches and the need to establish standards [36, 38]. The GRAP collaboration formed to further this approach and addresses aspects such as regulatory acceptability, the use of biological support data [37], and the applicability to nanomaterials or complex mixtures. The major advantage is that such an approach can actually be formally validated and uncertainties with any prediction can be quantified. The emergence of professional tools and services promises a much broader use of computational approaches both for REACH registration and other similar legislations worldwide, as well as for Green Toxicology practices.

### Predictive toxicology using omics and in vitro tools

In the initial stages of chemical development, the identification of the sequential processes and perturbations of biological pathways at a molecular level (e.g. molecular initiating events; MIE) through to the cellular or organ level leading to an adverse outcome provides insight into the SAR and can allow for the grouping of similar mechanisms of biological response [39]. These are the principles of adverse outcome pathways (AOPs), which can bridge the gap between responses on the cellular level to that of the whole organism, population, community and possibly ecosystem [39–42]. The use of toxicogenomic data and molecular techniques (i.e. omics; transcriptomics, proteomics and metabolomics) provides insight into mechanisms of action, and although not explicitly considered in regulatory decision making, omics data provide toxicological weight-of-evidence for the discovery and development of safe chemicals. Ankley et al. [42] outline a framework for AOPs, in which uncertainties and priorities associated with the toxicity of chemicals can be identified through either causal, mechanistic, inferential, or correlation based relationships; all derived from in vitro (including omics techniques), in vivo, or computational tests (e.g. QSARs, ToxCast, etc.). AOPs can help to improve across-chemical extrapolation and predictions of toxicity for chemicals that trigger the same MIE, which can be used to help design safer chemicals and products [15]. In addition, the adverse outcomes of in vitro tests can be compared with different levels of biological organization that are of regulatory relevance, which may reduce the need or reliance of live animal testing. However, the current level of detail of AOPs are mostly narrative and not quantitative, thus there is a need to move to more molecularly defined mechanisms, for which the term pathway of toxicity (PoT) has been coined [43], in order to allow for modelling. With PoTs, mechanism-based read-across studies will be feasible if all receptors within a potential pathway are examined in biological testing.

AOPs and PoTs will have major implications for the advancement of predictive toxicology, an important tool which would improve Green Toxicology approaches in developing and designing safer chemicals. An understanding of the molecular structure, functionality and adverse outcomes associated with chemicals is essential in aiding in the benign-by-design concept. In vitro assays are typically rapid, with high-throughput and large data output that can be reliably reproducible and cost effective. These qualities are particularly important for the safety and hazard assessment of new products, which can result in substantial costs for failures and problems detected late in the development and regulatory testing phase. In addition, many in vitro methods produce mechanism-specific data, which is another important aspect that can aid with the design of alternative compounds with lesser toxicity. With newer and more innovative in vitro tests, the focus moves more towards chronic exposures at low concentrations on cells and organ systems rather than the traditional adverse effects observed in in vivo animal tests that are often conducted at high doses. Furthermore, the results of in vitro testing (e.g. IC_50_ values in enzyme or receptor assays) could be used to develop predictive in silico tools. With the availability of such computational methods, chemists can test and screen their new structures for warnings, and learn to avoid the synthesis of toxic compounds at earlier stages of development. As such, the development of in vitro methods complement and may eventually reduce the essential use of in vivo studies, which directly supports the principles of Green Toxicology. Thus, with the addition of any new in vitro method for (eco)toxicity testing we will get one step closer towards an integrated process of gaining early toxicological information and adapting substance synthesis, leading to the efficient development of green chemicals and products with reduce toxicity.

The new approaches and scientific advances in molecular, cellular and computational toxicology can lead to a better application of predictive toxicology in the manufacturing of new materials and chemicals, which directly supports Green Toxicology. Predictive toxicology aims to develop new and innovative non-animal tests that do not simply duplicate existing animal tests but also provide a new scientific basis for product development and safety testing. Hence, the objective of such an application is to complement and extend traditional toxicity testing through a better understanding of toxicity pathways that contribute to products that are safer for humans and the environment. The incorporation and consideration of toxic potential and outcomes early in the design phase is an essential component of Green Toxicology and requires collaboration among chemists, toxicologists, industry, and regulators. Alternatives to current mandatory testing protocols are likely to influence future regulatory policy in Europe. However, if these new tools and methods for product development and risk assessment are to become widely accepted, then policy makers and regulators need to be informed and persuaded of the benefits of these alternative approaches and applications.

### Precautionary principle

Additional lessons for Green Toxicology can be learned from past product development and production through the application of the precautionary principle [17, 18]. The precautionary principle is applied in situations when harm to either the environment or human life does not need to be conclusively proven in order for risk to be addressed through discretionary decisions and policies. In some instances, the precautionary principle, often observed, is not distinguished from a “right”, which is respected and is a statutory requirement in some European Union member states [44]. In this respect, Green Toxicology can incorporate the precautionary principle by limiting the suspect or potential adverse toxicological consequences during the discovery, development and production of safer and more sustainable products. As such, knowledge about the previous selection of occupational, public health and environmental hazards can be examined to determine if further or earlier measures could have been employed to prevent harm (e.g. methyl tert-butyl ether replacement of lead in petrol; [16]). This concept is known as “late lessons from early warnings” and is often associated with the precautionary principle. Although often neglected, the precautionary principle offers many lessons and improvements that can be of use to developing products that are less harmful and should be a main driving force behind not only Green Toxicology but also Green Chemistry.

## Green Toxicology and animal testing: a chemical company’s perspective

Traditionally, the development of alternative testing methods in Europe was largely driven by ethical rationales such that studies were targeted for their use of many animals, or for their high potential to result in pain and suffering (e.g. skin and eye irritation testing). Regulatory rationales were also an additional driver of alternative test methods, particularly those that identified compounds with alerts for “cut-off” hazards, such as mutagenicity and endocrine disruption. Therefore, the currently validated in vitro assays particularly apply to the aforementioned endpoints [45, 46].

In the following paragraphs, a brief overview is provided concerning the 3Rs method currently employed at BASF, which are used within a screening context to avoid the development of compounds with an unfavourable hazard profile (i.e. concept of Green Toxicology).

### Skin and eye irritation studies

The traditional in vivo Draize irritation test for skin and eyes, in which a restrained, conscious animal is exposed (dermal and ocular, respectively) to a test substance for a set amount of time to determine toxicological effects, has long since been criticized for the limitations in species differences, subjective scoring, and experimental variability. The replacement of the Draize test for skin irritation was historically one of the first steps towards the full replacement of animal testing. BASF, and similar chemical companies, use two methods suitable to provide data for classification as corrosive (Epiderm™ skin corrosion test) or irritant (Epiderm™ skin irritation test) to the skin. These tests are employed within the context of a simple testing strategy described elsewhere [47–50]. In brief, a test substance is applied topically to a reconstructed human *epidermis* (RhE) that closely mimics the biochemical and physiological properties of the upper parts of the human skin using human derived non-transformed keratinocytes as cell sources. The indication of corrosive and irritant test substances is determined by their ability to decease cell viability (cytotoxicity) below defined threshold levels as measured via the MTT-assay.

For eye irritation, the situation is essentially quite similar. The replacement of the Draize test for eye irritation again was achieved by two methods. The ex vivo Bovine Corneal Opacity and Permeability (BCOP; [51, 52]) eye irritation test is used to identify strong eye irritation potential and the in vitro EpiOcular™ eye irritation test (EIT) is used to evaluate the irritation potential of compounds to the eye. The BCOP and similar ex vivo tests utilize slaughterhouse material to assess the severe eye irritation potential of a test substance through its ability to induce opacity and increased permeability in, for example, an isolated bovine cornea. In contrast, the EIT and similar tests use the commercially available reconstructed human cornea-like epithelium (RhCE), which closely mimics the histological, morphological, biochemical and physiological properties of the human corneal epithelium to determine if a test substance is an eye irritant based on its ability to induce cytotoxicity in RhCE tissue, as measured by the MTT assay. These tests, and alternatives, are again used within the context of a simple testing strategy and described in further detail elsewhere [52–56].

### Skin sensitization studies

Skin sensitization is a process more complex than skin or eye irritation, and includes several key events such as (1) dermal penetration, (2) protein reactivity, (3) inducing stress responses in keratinocytes, (4) activation of immune cells (dendritic cells) in the skin, and (5) their translocation to the lymph nodes. Given this complexity, it is difficult to imagine one single test that would be able to incorporate all of these steps [57]. Therefore, the development of an in vitro testing approach for skin sensitization resulted in the best solution [58], consisting of three assays addressing hazard identification according to the abovementioned key events 2–4. Protein reactivity is measured in the Direct Peptide Reactivity assay (DPRA) [59], stress responses are measured in Keratinocytes in either KeratinoSens [60, 61] or LuSens assays [62, 63], and immune cell activation is measured in the Human Cell Line Activation Test (h-CLAT) [64]. Empirical evidence for more than 200 compounds has shown that the best match with known human skin sensitizers is obtained by a “majority rule”, such that if two or more assays are positive, the compound is a skin sensitizer, while if two or more are negative, it is not [65]. With this testing strategy, a correlation with human skin sensitizers is obtained, which is slightly better than that obtained in the local lymph node assay (LLNA) [66, 67].

### Acute toxicity testing

The endpoint of systemic toxicity has not been of major interest to chemical companies and regulatory bodies. With good intention, it was proposed to use the results of cytotoxicity testing to determine the starting dose for acute oral toxicity testing. However, it should be noted here that good intention is not always a good guidance. With years of experience following this principle, a post validation study demonstrated that dose selection based on expert knowledge provided better results than following the cytotoxicity guidance, which was not significantly better than a fixed starting dose of 300 mg/kg b.w. [68]. Such expert knowledge was determined from information about the substance class or comparable formulation. Thus, Green Toxicology, which utilizes and considers all available information about a test substance through, for example, (Q)SAR, read-across and grouping of substances approaches may lead to reductions in the amount of chemicals and animals required for testing and development of new substances.

### Endocrine disruption

To screen for compounds with endocrine effects, two in vitro systems are often used that address the most common causes for endocrine activity: (1) agonist or antagonist effects on the androgen receptor (AR) or estrogen receptor (ER) and (2) interference with steroid synthesis. There are a variety of in vitro, wildlife and mammalian screen tests available to screen for endocrine disruptor activity, with details on each provided elsewhere [69]. In particular, the in vitro Yeast Estrogen Screen (YES)/Yeast Androgen Screen (YAS) assays are often used to screen for analyse effects on the AR and ER. The YES and YAS assays consist of yeast cell lines in which the human AR and ER have been introduced and coupled with a reporter gene that produces an enzyme. Activation and deactivation of either receptor are monitored by the change in colour of a dye sensitive to the activity of the enzyme. If deemed necessary, a follow-up is carried out at later stages of testing for endocrine activity with a refined 14- or 28-day study in which a blood metabolome analysis is included. Additional testing strategies for endocrine testing have been reported elsewhere [70, 71].

### Neurotoxicity

Another important aspect of systemic toxicity, with respect to avoidance of chemicals, with a problematic hazard profile is neurotoxicity. For screening purposes, the “neurons on a chip” assay is utilized [72, 73]. In this assay, primary neurons are grown on chips connected with a device that measures the spontaneous firing of the neurons. Compounds that stimulate or attenuate neuronal activity can be monitored by the changes in the firing rates of the neurons [74, 75].

### Developmental toxicity

The last, and possibly the most important endpoint in toxicology, which has been investigated in screening strategies, is the toxic effects on development. It should be noted here that relatively little is unfortunately known about the modes of action involved in developmental toxicity, in comparison with many endpoints in systemic toxicity such as neurotoxicity, endocrine effects or carcinogenicity. Therefore, mode of action-based screens are not readily available. Two more holistic approaches are used to assess developmental toxicity: (1) the chick embryotoxicity screening test (CHEST) [76] and (2) the fish embryo toxicity (FET) test [77, 78]. In both assays, the development of a complete embryo is monitored and evaluated during certain embryonic and foetal stages. As such, these tests are very close to being animal studies but are not considered as such because of the very early timing of testing and the absence of a maternal organism being exposed. A third test for assessing developmental toxicity is the mouse embryonic stem cell test (EST; [79–81]). In this test, the differentiation of pluripotent stem cells into cardiomyocytes are evaluated, and the interference with the normal differentiation process is a measure for the compound’s developmental toxicity potential [80]. It should be noted that all of the abovementioned tests are associated with certain limitations, such as over-sensitivity for acutely toxic compounds, over-sensitivity for compounds that are irritating/corrosive, lack of metabolism, water solubility, etc. Nevertheless, with the inclusion of additional endpoints, such as placental transfer (which can be measured in vitro), reasonable prediction values can be obtained for certain classes of chemicals [82]. However, it is clear that a better understanding of AOPs in developmental toxicity will be necessary to develop a series of targeted in vitro assays to entail better screening for this very important endpoint.

### New tools

With new omics technologies becoming more readily available, we are now at a point where there is a chance to tackle complex toxicological concerns, such as systemic toxicity. Following the successful development of a metabolomics based approach to predict systemic toxicity from a single drop of blood from short-term toxicity studies [83], the potential of this technology using an in vitro approach is being explored. A proof of principle was achieved using fibroblast to assess the effects of compounds on cell energy metabolism [84]. This work was followed by intensive research to establish an in vitro system combining liver cells and metabolomics for the identification of liver toxicity and modes of action. It has been observed that identification of liver toxicity can indeed be achieved by in vitro metabolomics using the HepG2 liver cell line. Research using cells from other organs is continuing; striving for the identification of organ-specific toxicity. The final goal would be to have a full array of cell systems to reliably predict systemic toxicity [85]. It is essential that work to obtain such alternative methods be continued because their availability is needed to reduce animal use, reduce the use and production of waste, and to increase the utility of such methods in chemical screening regulation. With this available, only a few grams of compound would be needed to evaluate systemic toxicity, and to move ahead one step further in the development of Green Chemistry with Green Toxicological methods.

Finally, with the availability of sufficient data on, for example, compound–receptor interactions, it is possible to create mathematical models which can assign a likelihood that a particular chemical structure will interact with a biological target. The process of developing such models is not necessarily fast and depends very much on the quality of the input data. However, the availability of such models will help chemists to design new compounds that perform the desired task and have a higher likelihood of a favourable toxicological profile. Safety-by-design will result in a win–win situation, with less animal testing and intrinsically safer products.

## Green Toxicology in drug development for human safety assessment

In contrast to household and consumer chemicals, where the optimization process of the properties during product development is often independent of the safety assessment, the drug development process can be seen as a series of iterative steps to optimize efficacy and simultaneously lower the safety as early as possible. Therefore, the early assessment of toxicity before the first application to man (clinical phase 1) plays a pivotal role in this process. Compounds for which the preclinical toxicological assessment identifies an adverse effect profile that exceeds the expected benefit for the patient will be excluded from progression in the development pipeline. Preclinical toxicology is hereby facing two challenges: on the one hand, the predictivity of the applied toxicological assays should be improved on a continuous basis to avoid false predictions (both false positives and false negatives), while on the other hand, the predictions should be made as early as possible during the process of drug candidate selection. This early assessment causes a shift from in vivo to in vitro to in silico methods. Maertens et al. [1] stress the parallels between the Green Toxicology movement and the strive for early and reliable safety assessment (“front-loading”) in the pharmaceutical industry, such that the achievements in meeting the abovementioned challenges will contribute to the objectives of Green Toxicology.

Some toxicological effects can in the meantime be predicted based on in silico methods with reasonable reliability, such as mutagenicity, phospholipidosis, and to a lesser extent skin sensitization [86]. It can be foreseen that integrated testing strategies will evolve with the advent of AOPs and a better understanding of the mechanisms of toxicological effects, which comprise a combination of in silico and in vitro tools to predict toxicological effects. For example, models that predict pharmacokinetic behaviour (absorption, distribution) of compounds based on physicochemical properties could be combined with predictions of liver transport based on QSAR transporter models. The inclusion of subsequent results from in vitro toxicity assays with hepatocytes or mitochondria will help to identify compounds that have a propensity towards drug-induced liver toxicity (DILI). Such complementary tools may limit and remove the most problematic candidates in early phases or allow medicinal chemistry departments to optimize the structure early on.

### Green Toxicology for the early assessment of environmental safety

Triggered by numerous publications on occurrence of pharmaceuticals in the environment, the European Commission was asked to deliver a strategic approach to pollution of water by pharmaceutical substances. The corresponding report was published in 2013 [87]. Regarding green medicinal products, the report concludes that “an approach to minimising the persistence, bioaccumulation and impacts of medicinal products on the environment would be to promote the replacement of substances of concerns by molecules with a more environmentally friendly profile or substances which demonstrated a higher rate of removal in wastewater treatment plants and to develop new compounds that are altogether effective, efficient and readily biodegradable in the environment.”

Despite these straightforward claims, the advances in the field of Green Toxicology for environmental safety are less evident than for human safety. The reason for this deficit is an inherent conflict of objectives during the optimization phase of a drug candidate, which is often overlooked in the discussion. One key criterion for low human toxicity is the partial stability of a drug candidate both with regard to human metabolism, as well as chemical stability towards light and temperature. Unless we consider a so-called pro-drug, which requires metabolic activation for achieving efficacy, an otherwise unstable compound usually undergoes attrition during the drug development. Degradation or rapid metabolism of a drug candidate usually results in a lower exposure to the efficacious compound leading to a lower efficacy of disease treatment. This lower efficacy could only be overcome by increasing the dose, which in turn could result in an increased risk of side effects. In addition, particular phase I metabolites or breakdown products may elicit adverse effects on their own, which can lower the therapeutic window.

Striving for optimization of drug stability may result in the persistence of the drug after excretion and in sewage treatment in the aquatic environment. For some drugs, the concentrations reported in certain aquatic environments raise the concern of causing harm to environmental species. As a matter of fact, the vast majority of active pharmaceutical ingredients show no ready biodegradability when subjected to the pertinent OECD screening tests for ready biodegradability [88]. Furthermore, there are currently no reliable tools or assays for predicting biodegradability in the early phases of drug development, which hinders the appropriate and desirable selection of biodegradable compounds. Two case examples are presented below to illustrate the described difficulties in the inclusion of Green Toxicology for environmental safety assessment.

#### Case example 1: the search for biodegradable iodinated X-ray contrast media

Iodinated X-ray contrast media are used to enhance the contrast between organs or vessels and surrounding tissues during radiography. After renal excretion, iodinated X-ray contrast media contribute to the burden of adsorbable organic halogens (AOX) in sewage water [89]. The high doses required to achieve radiocontrast can only be administered intravenously if the compounds are both stable and of extremely low toxicity. It comes as no surprise that optimized stability results in a low biodegradability (<10% in the test for ready biodegradability according to OECD 301) and that the compounds are detectable at microgram levels in sewage effluents and certain surface waters [89, 90].

**Fig. 2:**
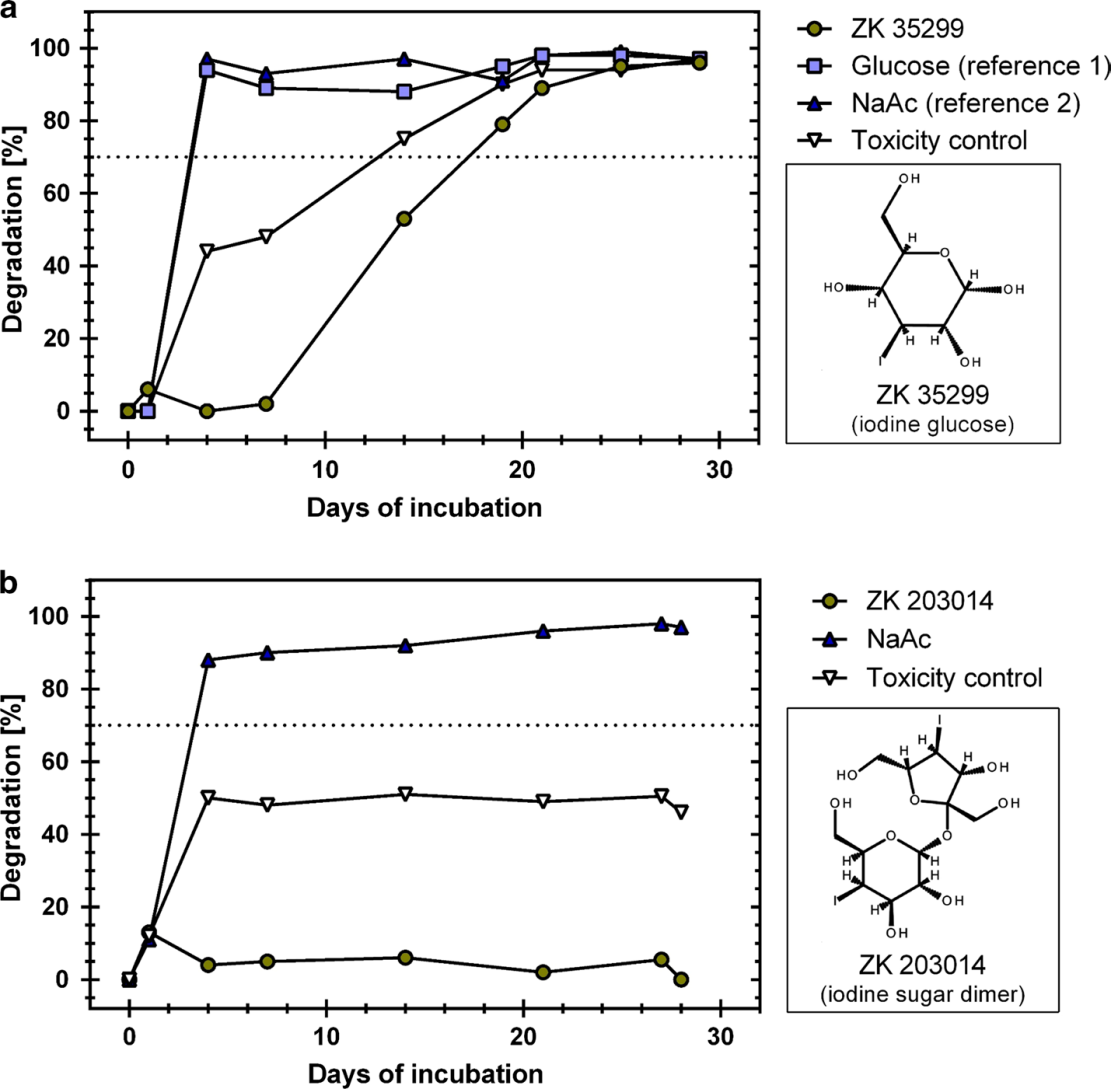
Degradation of iodinated sugar molecules. **a** Iodinated glucose is less readily degraded than its parent compound glucose or the reference compound sodium acetate (NaAc), but still reaches ready biodegradability within the 21-d window (*dashed line*). **b** The sugar dimer ZK 203014 with two iodines attached to achieve a higher radiocontrast shows only marginal biodegradability. In both graphs, the “Toxicity Control” depicts the result of the degradation of a 1:1 mixture of NaAc plus the test compound (ZK 35299 or ZK 203014). In both cases, these curves approximate the combination of the individual degradation curves of NaAc and the test compound, indicating that the test compound does not inhibit the degradation of NaAc by microcidal action

In an effort to find alternative chemical core structures capable of carrying the radio-dense iodine, while at the same time being better biodegradable in the aquatic environment, iodine sugars were investigated as candidates for X-ray contrast media (Fig. 2). A single iodine bound to a monomeric sugar molecule (iodo-glucose) fulfilled the criterion of being readily biodegradable (>70% degradation within 21 days). However, the attachment of only one iodine atom per sugar molecule was not sufficient to achieve the required radiocontrast at a feasible dose. Therefore, alternative sugar dimers carrying two iodine atoms were investigated, but these showed a significant decrease in the degradability. In addition, the toxicity of the iodine sugar dimer proved to be 8.3 times greater compared with the conventional contrast media in rodent studies. Furthermore, the sugar dimer showed low heat stability (i.e. it was not amenable to heat sterilization), which is a prerequisite for an injectable compound, if costly filtration sterilization is to be avoided. Due to these significant drawbacks, the chances of success in the search for alternative structures were considered to be inherently low, and the programme was subsequently stopped.

#### Case example 2: glufosfamide—a model compound for benign-by-design?

The two oxazaphosphorines, ifosfamide and cyclophosphamide, belong to the most frequently used antineoplastic agents in cancer therapy. Due to their mutagenic and carcinogenic potential, concern was raised that the compounds might occur in the environment after excretion and cause harm to aquatic species. Both compounds showed only minor biodegradability in laboratory-scale sewage treatment plants [91].

**Fig. 3:**
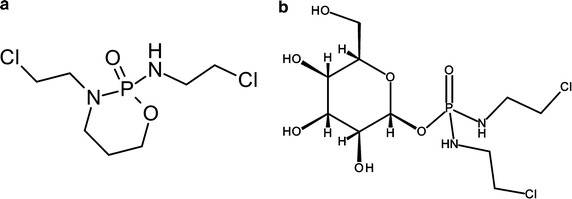
Structures of the marketed alkylating agent ifosfamide (**a**) and its glucose derivative, glufosfamide (**b**)

In 2000, Kümmerer et al. [92] published biodegradability data for the cancer drug candidate glufosfamide. Glufosfamide is an alkylating agent closely related to ifosfamide, in which the AI of the oxazaphosphorines (isophosphoramide mustard) is linked to β-d-glucose by an *O*-glycosidic bond (Fig. 3). The intention of this modification is primarily to utilize the overexpression of glucose transporters (GLUT) in tumour cells for increased cellular uptake of the cytotoxic agents into cancer cells [93], thereby augmenting the efficacy of this alkylating drug candidate. Glufosfamide showed a significantly higher biodegradability compared with ifosfamide or cyclophosphamide; however, the criterion for ready biodegradability according to the OECD 301 guidelines was also not achieved. A paper by Kümmerer [94] later postulated that this chemical modification could be taken as an example for the “benign-by-design” paradigm since this molecule should not only benefit the patient but also show advantageous environmental properties.

Unfortunately, and despite encouraging preclinical results pointing towards lower toxicity of glufosfamide compared with ifosfamide, several clinical trials have not resulted in the approval of the potential drug. However, orphan drug status was granted for glufosfamide for pancreatic cancer by both the U.S. Food and Drug Administration (2006) and the European Medicines Agency (2011) [95]. Clinical Trials [96] list nine studies with glufosfamide between April 2000 and Sept 2013 for a broad variety of cancers. Six of these studies are reported as “completed”, two as “terminated”, and one is still recruiting in the indication pancreatic cancer.

A review by Calvaresia and Hergenrother [97] discusses the high dose-limiting human toxicity towards erythrocytes compared with the approved drug ifosfamide, with the main cause for the lack of clinical success of this compound stated as: “… the anemia observed may stem from the fact that erythrocytes express high levels of GLUT. Clinical testing of future glucose conjugated drugs should be cognizant of this potential hemolytic phenotype”. Evidently, the glucose modification of the cytotoxic agents increase human toxicity rather than increase the efficacy of the drug, which contrasts with published preclinical results [97]. Thus, it is doubtful whether sugar attachment can be considered as a general design feature for degradable drugs due to the toxicity liabilities that such a chemical modification might introduce.

Concluding the two presented case studies, there are currently no straightforward strategies available for the inclusion of Green Ecotoxicology into the early phases of drug development.

## Green Toxicology for nanomaterials: read-across and grouping as tools for predictivity

Over the last two decades, nanomaterials are more and more in the focus of scientists, production companies, but also of regulators. This family of relatively new compounds and materials is different from the normal definition of chemical compounds. Chemical substances are usually described by their chemical composition but in the case of nanomaterials, additional descriptors such as particle size, shape or composition of core and coatings are needed to specify and distinguish them from each other. As a consequence, a virtually unlimited number of different nanomaterials can be identified, which may result in a burdensome request for a large amount of toxicological data for regulatory hazard assessment. It is important to ensure that the development of new nanotechnology occur in the presence of Green Toxicology and Chemistry practices (e.g. focus on preventive design). A framework for chemists and material developers is needed to clearly outline design rules that integrate health, safety, and environmental concerns into nanotechnology development [98]. Thus, for Nanotechnology as a relatively young technology, the opportunity exists to start early on with the implementation of the principles of Green Toxicology. However, as highlighted by Hansen et al. [98] research on the sustainability of materials must be funded at levels significant enough to identify early warnings, and regulatory systems should provide incentives for safer sustainable materials. Two examples of common nanotechnology are presented below that emphasize the complexity of early warning identification and integration of Green Toxicology practices.

### Case 1: metal oxides such as titanium dioxide as inert substitutes

Cosmetics, especially sunscreens, should protect us from ultraviolet (UV)-light induced sunburn and skin cancer. This protection has been achieved by a multitude of chemicals with different structures, some of which are under suspicion of being endocrine disruptors or of having other effects in environmental organism in receiving aquatic environments. Over the last two decades, nanoparticles consisting of ZnO or TiO_2_ have been used as very efficient physical UV-blocking materials. As the natural background for TiO_2_ is relatively high in surface water, such as lakes and rivers [99], the use of TiO_2_ as a UV-blocking agent is less hazardous than the “normal” chemical cocktail in sun creams. However, recently an intense discussion was started on the possible carcinogenic effect in the lung after inhalation of sun screens, as the International Agency for Research on Cancer (IARC) stated: “Titanium dioxide is [a] *possible carcinogenic to humans (Group 2B)* based on sufficient evidence in experimental animals and inadequate evidence from epidemiological studies” [100]. This example brings together considerations about a product that has been on the market for decades, despite outcomes of experiments describing relatively severe effects in cells or animals. The idea of Green Toxicology may help to resolve this problem by introducing specific information about the materials used and by establishing relationships between the properties of the TiO_2_-particles and the predicted outcomes. Comparisons of the materials used for the critical animal studies with that produced for the sunscreens should allow for the determination of the similarities in the materials and if the benign-by-design principle should be considered more thoroughly for future development of sunscreens.

### Case 2: carbon nanotubes—possible carcinogens but degradable?

Another critical nanomaterial is carbon nanotubes (CNTs), a lightweight but very strong material with a multitude of different possible applications in its various modifications (i.e. single-walled and multi-walled CNTs with different conformations). This material is described to have a strong similarity to asbestos with respect to the adverse health effects caused via inhalation. The properties of CNTs, such as the biopersistent, long fibre-like structure, and induction of oxidative stress, lead to the same biological consequences in lung tissue as asbestos, and thus, the use of CNTs is still under debate regarding the carcinogenic effect. However, following the important principle “benign-by-design”, recent studies have demonstrated that CNTs are biodegradable under specific circumstances, which would dramatically reduce their carcinogenic potency [101, 102]. In addition, it has recently been demonstrated that short fibre lengths less than 5 µm are not severely toxic and that only long and rigid CNTs have an asbestos-like effects [103]. Hence, this case study is an example of a situation where toxicological information for the production of benign CNTs already exists and should be used for future development of products.

### Nanomaterials—endless variability needs new tools for assessment

As mentioned above, more and more products that consist of or contain nanomaterials will soon enter the market or are already in use. An adequate risk assessment of environment and health is seemingly not possible because of the tremendous need for biological experiments, animal testing and laboratory capacity. The usefulness and applicability of in vitro methods must be demonstrated on a case-by-case basis, but they represent enabling technologies to address these demands [104, 105]. There are also opportunities for in silico approaches and pragmatic solutions such as grouping and thresholds of toxicological concern. Thus, the concepts of read-across and grouping, which are described in detail for chemicals by the OECD and ECHA, should also be introduced for nanomaterials. In short, read-across is defined by ECHA as “the use of relevant information from analogous substances (the ‘source’ information) to predict properties of the ‘target’ substance(s) under consideration” [106]. As mentioned earlier, the starting position for this approach is the formation of groups of chemicals or materials which have the same properties for a specific aspect. The OECD defines this approach as follows, “the term ‘grouping’ or ‘chemical grouping’ describes the general approach for considering more than one chemical at the same time” [107]. Thus, a grouping of nanomaterials combined with a corresponding evaluation and test strategy based on, for example, their physicochemical properties or toxicological characteristics, would reduce regulatory testing efforts. This has already been recognized early on and several grouping frameworks have been proposed [108–113]. The concept of Walser et al. [111] proposes as the first step to group the unlimited nanomaterials identified into a limited number of entities. The chemical composition of each structural element (core, coating, etc.), size distribution, and shape are subdivided into predefined toxicologically relevant bands, wherein read-across criteria can be applied. These biunique entities may include many similar nanomaterial identities, which are considered the same from a regulatory perspective. In a second step, entities are allocated to groups (clouds), which represents specific testing strategies for toxicological endpoints that need further evaluation [111]. This allocation is driven by AOPs [27], in which key events are triggered in a cascade-like manner, ultimately leading to an undesirable biological response [114, 115].

In addition to hazard-oriented key events, properties such as stability or bioaccumulation can serve as further building blocks for testing strategies [116]. Bio-persistent accumulative entities of nanomaterials, capable of inducing key events responsible for long-term toxicity, would be allocated to a testing strategy where further testing is needed. In contrast, nanomaterials that are not triggering such key events would be allocated to a group where no such additional testing is required. The assignment of substances and nanomaterials to predefined testing strategies based on AOPs further support Green Toxicology. Data on the induction of key events of relevant AOPs could be screened by in vitro assays and serve as gatekeepers in innovation processes.

## Conclusions

The cases shown above for chemical and pharmaceutical companies, as well as nanotechnology development, clearly demonstrate that Green Chemistry, together with the principles of Green Toxicology more specifically related to the environmental and health effects of compounds or materials, may achieve a sustainable and safe production scenario of new chemicals. However, in the case of pharmaceutical compounds, there may also be limitations with regard to achieving safe and efficacious drugs that are at the same time environmentally friendly. As the examples from the European Environmental Agency (EEA) demonstrate [16], it is now the duty of all the stakeholders to implement such rules for a responsible production of new compounds and materials based on common principles. Taking the ideas of Maertens et al. [1] as a basis, the principles of Green Toxicology may be further expanded. It is not only important to test early, but to also try to achieve safety-by-design of the compounds, to use predictive test systems, and to avoid exposure. Overall, testing itself must be sustainable and safe by avoiding solvents that may be hazardous or energy consuming, and testing should help to reduce the need of experimental animals. Moreover, the ideas and fundamental rules of toxicology should be familiar for all chemists but also to physicists and engineers. Thus, a transdisciplinary education in toxicology would be helpful to implement this knowledge in the processes for chemical development (Fig. 1).

Last but not least, such measures are not free of charge, hence, all stakeholders should be convinced to use these principles and the consumer has to accept the higher costs for such products. As a very important step in producing chemicals and materials for future applications regarding environmental- and health-safety issues, the goals of Green Toxicology have to be accepted by all societal groups.

## Abbreviations

AI: active ingredient
AOX: adsorbable organic halogens
AOP: adverse outcome pathway
BCOP: Bovine Corneal Opacity and Permeability
CNTs: carbon nanotubes
CHEST: chick embryotoxicity screening test
DPRA: Direct Peptide Reactivity assay
DILI: drug-induced liver toxicity
EST: embryonic stem cell test
EIT: eye irritation test
ECHA: European Chemical Agency
CEFIC: European Chemical Industry Council’s
EEA: European Environmental Agency
GLUT: glucose transporters
FET: fish embryo toxicity
GLP: Good Laboratory Practice
GRAPs: Good Read-Across Practices
h-CLAT: Human Cell Line Activation Test
IARC: International Agency for Research on Cancer
LLNA: local lymph node assay
MIE: molecular initiating event
OECD: Organisation for Economic Co-operation and Development
PoT: pathway of toxicity
PAHs: polyaromatic hydrocarbons
PCBs: polychlorinated biphenyls
PET: polyethylene terephthalate
PMI: process mass intensity
QSARs: quantitative structural activity relationships
REACH: Registration, Evaluation, Authorisation and Restriction of Chemicals
SAR: structural-activity relationships
UV: ultra-violet
VOC: volatile organic carbons
YAS: Yeast Androgen Screen
YES: Yeast Estrogen Screen
